# Density of ectopic fat depots predict distinct biomarkers of glycemic and insulinemic status in persons with HIV

**DOI:** 10.1038/s41387-025-00381-y

**Published:** 2025-06-18

**Authors:** Seungweon Park, Annaliese Widmer, Alison Z. Swartz, John R. Koethe, Heidi J. Silver

**Affiliations:** 1 Vanderbilt University, School of Medicine, Nashville, TN USA; 2 Vanderbilt University Medical Center, Department of Medicine, Division of Gastroenterology, Hepatology and Nutrition, Nashville, TN USA; 3 Vanderbilt University Medical Center, Department of Medicine, Division of Infectious Diseases, Nashville, TN USA; 4 Tennessee Valley Healthcare System, Department of Veterans Affairs, Nashville, TN USA

**Keywords:** Risk factors, Diseases

## Abstract

**Background:**

HIV and obesity are conditions of impaired lipid storage where ectopic lipid accumulates in organs and tissues, promoting glucose intolerance and insulin resistance. Persons with HIV (PWH) are at high risk for diabetes, and one indicator of risk is the density of organs and tissues involved in glucose metabolism, which reflects ectopic lipid content and can be quantified using CT-tissue attenuation. We investigated relationships between subcutaneous adipose (SAT), visceral adipose (VAT), liver, pancreas, and skeletal muscle densities with biomarkers of glycemic/insulinemic status.

**Methods:**

Demographic, anthropometric, and clinical data were utilized with automated segmentation of CT morphometric data from images acquired at the 3rd lumbar vertebra level in PWH who had normoglycemia, prediabetes, and T2DM.

**Results:**

Of 217 PWH, 29.0% had prediabetes and 30.4% had T2DM. Liver, pancreas, and skeletal muscle densities were lower, and SAT density was higher, in PWH with T2DM. No differences were observed for VAT density. Receiver operating curves adjusted for age, sex and BMI showed tissue densities had similar ability to discriminate glycemic/insulinemic status. Adjusted multivariable logistic regression showed higher SAT density associated with higher glucose (*p* = 0.002), HbA1c (*p* < 0.001), and diabetes status (*p* < 0.001). Lower liver density is associated with diabetes status (*p* = 0.007) and higher HbA1c (*p* = 0.03), whereas lower skeletal muscle density is associated with higher glucose (*p* = 0.03) and insulin (*p* = 0.04).

**Conclusions:**

Tissue densities, which differed significantly among the three groups, were robustly associated with various biomarkers of glycemic/insulinemic status. CT-morphometrics may enhance the detection of metabolic perturbations and diabetes risk, possibly earlier than some clinical biomarkers.

## Introduction

With improved understanding of HIV disease pathophysiology and advancements in the efficacy of anti-retroviral treatment (ART), life expectancy of people with HIV (PWH) has increased substantially over the past three decades [1]. Concomitantly, the incidence and prevalence of progressive chronic diseases, including type II diabetes (T2DM), have risen. Compared to the general population of U.S. adults, PWH are estimated to have a 3.8–5.0% greater prevalence of T2DM [2]. While traditional risk factors contribute to the development of T2DM in PWH, there appears to be an exaggerated risk from weight gain [3–5]. For example, the Veterans Aging Cohort Study showed a 14% increased risk for T2DM with each five pounds of weight gained in PWH compared to an 8% increased risk in individuals without HIV [6].

The increased risk for T2DM also derives, in part, from the deposition of “ectopic” fat within and around the heart, abdominal organs, and skeletal muscle, which impairs normal physiologic functioning and occurs with greater frequency and severity in PWH [7]. The redistribution of adiposity is linked to impaired ability of subcutaneous adipose tissue (SAT) to store excess lipids as triglycerides, resulting from local effects of viral proteins and changes in the SAT immune environment that also promote increased accumulation of metabolically unhealthy visceral adipose tissue (VAT) [8]. Hence, both high whole body and visceral adiposity are now common in treated HIV [9]. Accumulation of excess VAT is associated with dyslipidemia, systemic inflammation, and insulin resistance [10]. Further, a higher VAT to SAT ratio independently predicts cardiovascular events [11], the leading cause of mortality in PWH.

The development of computed tomography (CT) imaging to distinguish and quantify adipose and muscle tissue areas has revealed complex relationships between distinct adipose tissue depots and cardiometabolic health outcomes. Beyond identifying the impact of the amounts of tissues, morphometric segmentation of CT images has enabled investigation of the density of organs and muscle based on radiographic attenuation, which reflects ectopic fat content [12]. Introduction of ART in treatment-naive PWH has led to reduced SAT and VAT densities [13]. Reduced adipose tissue density reflects a higher ratio of lipids compared to the more dense cellular material and stromal structures, and thus, is associated with greater systemic inflammation and higher triglyceride to HDL-cholesterol ratio, indicating greater insulin resistance [14]. Further, the density of liver quantified by CT attenuation value has been used to determine the prevalence and metabolic impact of hepatic steatosis in PWH [15].

In addition to greater cardiometabolic disease risk, PWH are more vulnerable to a rapid decline in skeletal muscle, physical conditioning, and the development of frailty as they age [16]. Low skeletal muscle density increases the risk for sarcopenia and consequent impairment in physical functioning [17]. Indeed, CT-based measurements show thigh muscle density is lower in males with HIV compared to HIV-negative males [18], and reduced density of thigh and trunk muscles is associated with slower and weaker measures of physical function [19]. The infiltration of ectopic fat into skeletal muscle occurs both between muscle groups (intermuscular adipose tissue) and within muscle fibers (intramyocellular lipid). As skeletal muscle is the primary site of insulin-stimulated glucose disposal, the condition of excess inter- and intramyocellular lipid, and thereby low skeletal muscle density (termed myosteatosis), also promotes insulin resistance and predicts incident T2DM [20, 21].

There remains a paucity of data on relationships between individual ectopic fat depots and biomarkers of cardiometabolic health among individuals on contemporary ART. A lower density represents enlarged, lipid-overloaded, poorer quality adipocytes in PWH. A key question is whether the density of organs and tissues predicts hyperglycemia and insulin resistance [22]. Identifying morphometric features of PWH who are at high cardiometabolic risk would enable the design of tissue site-specific interventions to prevent the onset or progression of overt metabolic disease. The objectives of this study are threefold: (1) to identify differences between PWH with no diabetes, prediabetes, and T2DM for an array of CT-quantified morphometric features (SAT area, SAT density, VAT area, VAT density, skeletal muscle area, skeletal muscle density, pancreas density, liver density) along with traditional clinical biomarkers; (2) to determine specific relationships between CT-quantified tissue densities and glycemic/insulinemic status; and (3) to evaluate the performance of CT-quantified tissue densities as indictors of glycemic/insulinemic status in PWH.

## Methods

### Ethics approval

The study was approved by the Institutional Review Boards of the Vanderbilt University Medical Center (VUMC) IRB# 191911 and the Tennessee Valley Healthcare System (TVHS) IRB# 1488525-2, and all participants signed written informed consent.

### Participants

This study utilizes baseline data from two prospective cohorts of adults with treated HIV. The first cohort was recruited from the Comprehensive Care Clinic at VUMC between August 2017 and November 2019. The second cohort was recruited from the Infectious Diseases Clinic of TVHS between November 2020 and December 2022. Inclusion criteria were age ≥21 years and use of one ART regimen for >6 months with HIV-1 RNA <50 copies/mL and CD4+ count >350 cells/µL at enrollment. Prediabetes and diabetes status were determined by the most recent ICD-9/10 diagnosis codes, HbA1c, and/or medication treatment. Exclusion criteria were cirrhosis, active hepatitis B or C, other inflammatory or rheumatologic conditions, treatment with insulin or DPP-4 inhibitors, tesamorelin, oral corticosteroids, hormone replacements, illicit drug use, or alcohol intake >2 drinks/day. Of note, no participants were receiving GLP-1 analogs or SGLT-2 inhibitors.

### Body composition/morphometrics

Anthropometric data (height, weight, and waist circumference) were measured in triplicate and averaged. An automated version of Slice-O-Matic software (Version 4.3, TomoVision, Montreal, Canada) was used to quantify abdominal SAT, VAT, and skeletal muscle areas and radiodensities from non-contrast CT images of the abdomen/pelvis at the level of the third lumbar vertebra. Liver and pancreas attenuation were determined by a clinical radiologist using methods previously described [23]. Liver attenuation ≤42 Hounsfield units (HU) indicates moderate to severe hepatic steatosis with ≥26% cellular fatty infiltration. Skeletal muscle index (SMI) was calculated by dividing skeletal muscle area (SMA) by height (cm^2^/m^2^). Participants were considered to have sarcopenia if SMI was <43 cm^2^/m^2^ with BMI < 25 kg/m^2^ or <53 cm^2^/m^2^ with BMI ≥ 25 kg/m^2^ in males and SMI < 41 cm^2^/m^2^ in females with any BMI. Male and female participants were considered to have myosteatosis if skeletal muscle density (SMD) was <41.0 HU with BMI < 25 kg/m^2^ or <33.0 HU for BMI ≥ 25 kg/m^2^ for males and females [24].

### Clinical biomarkers

Blood samples were collected from participants after 10–12 hours in the fasted state and assayed at the VUMC Analytical Services Core for glucose, insulin, and c-peptide measurements. HOMA-IR score was calculated according to the formula: fasting insulin (mU/L) × fasting glucose (mg/dL) divided by 405. Serum for lipid profiles was assayed at the VUMC and TVHS pathology laboratories, with assays from the labs showing equivalent upper and lower detection limits. Plasma for cytokine analysis was assayed at the VUMC Ware Lab using MSD V-PLEX Human Cytokine Immunoassay Kits (Meso Scale Diagnostics, Rockville, MD).

### Statistical analysis

A priori sample size analysis showed that with 45 persons per group, we have 80% power to detect a mean difference of ≥4.1 HU in skeletal muscle density between those with diabetes versus those with prediabetes or no diabetes. Variables were assessed for normality using visual inspection of histograms created with MATLAB version R2021a. Variables that did not display normality were log-transformed. Means and standard deviations were calculated for continuous variables, and numbers and frequencies for categorical variables. Spearman’s rho correlations were performed to determine associations between dietary, metabolic, and CT-quantified organ and tissue variables. Multiple imputation was performed for missing values using the R MICE package in R Studio with R version 4.3.2 (R Foundation for Statistical Computing, Vienna, Austria). The ability of CT-quantified organ and tissue (VAT, SAT, liver, pancreas, skeletal muscle) densities to distinguish glycemic/insulinemic biomarkers was assessed using receiver operating characteristics (ROC) to quantify and compare the area under the curves (AUC) of tissue densities for each individual biomarker. To improve the value of information from comparing tissue densities, ROC analysis was adjusted for age, sex and BMI. Predictive performance was further evaluated using multivariable logistic regression models adjusted for age, sex and BMI for each of the tissue densities separately with five biomarkers of glycemic status (diabetes status no/yes, HbA1c ≥5.7%, fasting glucose ≥100 mg/dL, fasting insulin ≥12.5 mIU/L, and HOMA-IR score >2.5) as dependent variables. The Hosmer-Lemeshow goodness-of-fit test was used to test the quality of the overall fit of the individual logistic regression models. Finally, to determine the best fit of all the morphometric data (amounts and densities), Akaike information criterion forward-backward stepwise regression was used to remove nonsignificant demographic, clinical, and morphometric covariates while achieving the most parsimonious model for each biomarker of glycemic/insulinemic status. The variance inflation factor was capped at five to reduce potential multicollinearity.

## Results

### Differences in demographic and HIV characteristics

Of the 217 participants (187 males and 30 females), 29.0% had prediabetes and 39.4% had T2DM (Table 1). On average, participants with diabetes were older than those with prediabetes and those with normoglycemia (56.3 ± 11.2 yrs vs 50.8 ± 12.1 and 47.2 ± 11.9 yrs, respectively, *p* < 0.001), had higher CD4:CD8 ratio (*p* = 0.04), had higher BMI (34.4 ± 7.4 kg/m^2^ vs 32.5 ± 6.8 and 30.5 ± 6.6 kg/m^2^, respectively, *p* < 0.001), and had a slightly longer duration of HIV (18.2 ± 9.9 *vs* 16.2 ± 9.3 and 14.5 ± 9.2 yrs, respectively, *p* = 0.09). The proportion of integrase strand transfer inhibitor (INSTI)-based ART regimens did not differ among the three glycemic status groups (*p* = 0.67).

**Table 1:** Demographic, clinical, and morphometric characteristics of 217 adults with treated HIV.

	No diabetes (*n* = 88)	Prediabetes (*n* = 63)	Diabetes (*n* = 66)	*p* value
**Demographics**				
Age (yrs)	47.2 ± 11.9	50.8 ± 12.1	56.3 ± 11.2	<0.001
Sex is Male	78 (88.6%)	55 (87.3%)	55 (83.3%)	0.55
Race is Black	42 (47.7%)	31 (49.2%)	32 (48.5%)	0.45
HIV duration (yrs)	14.5 ± 9.2	16.2 ± 9.3	18.2 ± 9.9	0.09
INSTI (current usage)	70 (79.5%)	50 (79.4%)	54 (81.8%)	0.67
**Clinical biomarkers and cytokines**				
CD4 (count)	774.5 ± 294.8	783.2 ± 319.4	857.2 ± 378.3	0.32
CD4 (%)	35.1 ± 8.0	32.4 ± 10.1	36.8 ± 11.5	0.05
CD8 (count)	852.5 ± 336.3	946.8 ± 441.5	854.5 ± 383.6	0.31
CD4:CD8 (ratio)	1.00 ± 0.42	0.98 ± 0.57	1.27 ± 0.99	0.04
HbA1c (%)	5.2 ± 0.4	5.9 ± 0.2	7.5 ± 1.8	<0.001
Glucose (mg/dL)	88.8 ± 6.9	110.2 ± 12.4	167.1 ± 72.1	<0.001
Insulin (μU/mL)	15.8 ± 15.8	36.1 ± 41.5	42.2 ± 27.8	<0.001
C-Peptide (ng/mL)	3.0 ± 1.4	3.9 ± 2.1	4.6 ± 1.2	0.01
HOMA-IR (score)	3.4 ± 3.2	9.8 ± 11.1	17.7 ± 15.9	<0.001
Total cholesterol (mg/dL)	173.0 ± 33.4	181.9 ± 40.2	166.9 ± 36.6	0.09
HDL (mg/dL)	44.8 ± 15.6	43.6 ± 15.8	40.4 ± 10.3	0.17
LDL (mg/dL)	106.3 ± 30.7	113.0 ± 39.7	95.5 ± 30.9	0.005
Triglycerides (mg/dL)	123.9 ± 68.5	154.6 ± 86.6	189.1 ± 163.9	0.003
TG/HDL (ratio)	3.38 ± 2.95	4.09 ± 2.81	5.75 ± 9.34	0.005
C-reactive protein (mg/dL)	3.5 ± 3.1	4.6 ± 5.3	6.6 ± 12.3	0.04
IL-1β (pg/mL)	0.22 ± 0.09	0.27 ± 0.37	0.31 ± 0.10	0.27
IL-12p70 (pg/mL)	0.28 ± 0.15	0.26 ± 0.19	0.29 ± 0.23	0.22
IL-6 (pg/mL)	1.09 ± 0.88	1.29 ± 0.97	1.59 ± 1.01	0.01
IL-8 (pg/mL)	6.27 ± 4.16	6.14 ± 3.62	7.17 ± 3.43	0.23
IL-10 (pg/mL)	0.72 ± 1.71	0.69 ± 1.41	0.44 ± 0.36	0.17
INF-ɣ (pg/mL)	9.92 ± 12.46	9.88 ± 13.09	9.45 ± 13.53	0.75
TNF-α (pg/mL)	1.42 ± 0.21	1.46 ± 0.54	1.49 ± 0.55	0.01
**Body composition and morphometrics**				
Height (cm)	175.5 ± 8.4	174.6 ± 8.9	174.4 ± 8.8	0.67
Weight (kg)	93.6 ± 13.1	99.3 ± 20.2	103.8 ± 20.1	0.002
BMI (kg/m^2^)	30.5 ± 6.6	32.5 ± 6.8	34.4 ± 7.4	<0.001
Waist circumference (cm)	100.9 ± 13.4	107.1 ± 13.2	115.9 ± 13.8	<0.001
Waist/hip (ratio)	0.95 ± 0.07	0.97 ± 0.07	1.02 ± 0.08	<0.001
SAT area (cm^2^)	249.7 ± 111.9	271.5 ± 122.5	310.9 ± 170.5	0.03
SAT density (HU)	−101.4 ± 6.3	−101.2 ± 5.0	−98.9 ± 6.9	0.02
VAT area (cm^2^)	185.9 ± 87.4	229.0 ± 97.4	280.5 ± 106.7	<0.001
VAT density (HU)	−97.3 ± 6.8	−98.2 ± 6.3	−98.2 ± 3.7	0.32
VAT/SAT (ratio)	0.89 ± 0.80	1.09 ± 0.89	1.22 ± 0.82	0.04
Skeletal muscle area (cm^2^)	188.9 ± 33.5	191.6 ± 38.9	184.1 ± 34.8	0.51
Skeletal muscle density (HU)	40.3 ± 5.3	38.1 ± 7.4	33.7 ± 7.2	<0.001
Liver density (HU)	59.3 ± 9.2	57.9 ± 9.2	53.5 ± 11.8	0.004
Pancreas density (HU)	38.2 ± 11.8	38.5 ± 11.3	35.1 ± 11.3	0.03

^a^No participants with diabetes were being treated with insulin.

### Differences in glycemic, lipid, and inflammatory profiles

As expected, participants with diabetes had worsened markers of glucose tolerance and insulin resistance than those with prediabetes or no diabetes, with higher HbA1c (7.5 ± 1.8 vs 5.9 ± 0.2 and 5.2 ± 0.4, respectively, *p* < 0.001), fasting glucose (167.1 ± 72.1 mg/dL vs 110.2 ± 12.4 and 88.8 ± 6.9 mg/dL, respectively, *p* < 0.001), fasting insulin (42.2 ± 27.8 μU/mL vs 36.1 ± 41.5 and 15.8 ± 15.8 μU/mL, respectively, *p* < 0.001), fasting c-peptide (4.6 ± 1.2 ng/mL vs 3.9 ± 2.1 and 3.0 ± 1.4 ng/mL, respectively, *p* = 0.01), and HOMA-IR score (17.7 ± 15.9 vs 9.8 ± 11.1 and 3.4 ± 3.2, respectively, *p* < 0.001). Participants with diabetes also had higher serum triglyceride levels (189.1 ± 163.9 mg/dL, *p* = 0.003) and higher TG:HDL ratios (5.75 ± 9.34, *p* = 0.005). Higher serum C-reactive protein levels (6.6 ± 12.3 mg/dL, *p* = 0.04) along with higher levels of pro-inflammatory cytokines IL-6 (1.59 ± 1.19, pg/mL, *p* = 0.01) and TNF-α (1.49 ± 0.55 pg/mL, *p* = 0.01) indicated greater systemic inflammation in participants with diabetes.

### Differences in body composition/CT morphometrics

Both waist circumference and waist-to-hip ratio were significantly higher in participants with diabetes (*p* < 0.001 for both) compared to those with prediabetes and no diabetes. Participants with diabetes also had higher amounts of SAT (310.9 ± 170.5 cm^2^ vs 271.5 ± 122.5 and 249.7 ± 111.9 cm^2^, respectively, *p* = 0.03) and VAT (280.5 ± 106.7 cm^2^ vs 229.0 ± 97.4 and 185.9 ± 87.4 cm^2^, respectively, *p* < 0.001). SAT density was also highest in those with diabetes (−98.9 ± 6.9 HU, *p* = 0.02), but no difference was observed in VAT density among the three groups (*p* = 0.32). In contrast, the densities of the pancreas (35.1 ± 11.3, *p* = 0.03), the liver (53.5 ± 11.8, *p* < 0.001) and skeletal muscle (33.7 ± 7.2 HU, *p* < 0.001) were lowest in participants with diabetes and the proportion of those with hepatic steatosis was greatest in those with diabetes (28.3% vs 11.9 and 10%, respectively, χ^2^ = 8.92, *p* = 0.01). While no significant difference was observed between groups in the proportion of participants categorized as having sarcopenia (15.0%, 11.7% and 7.4%, respectively, χ^2^ = 1.90, *p* = 0.39), significantly more participants with diabetes met criteria for myosteatosis (46.7% vs 23.3 and 5.9%, respectively, χ^2^ = 28.75, *p* < 0.001).

### Relationships between tissue densities and glycemic/insulinemic status

**Fig. 1:**
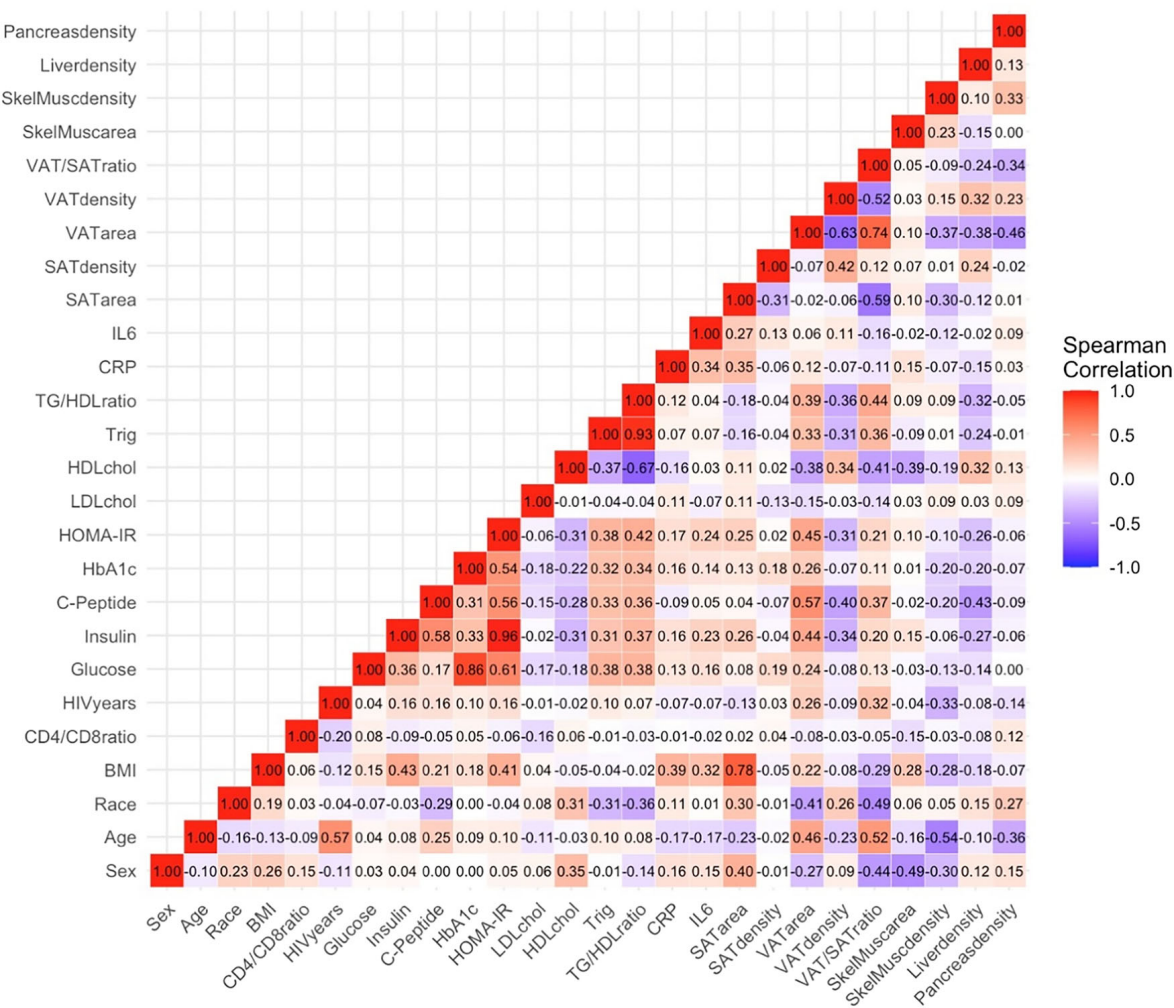
Heatmap displaying univariate relationships between demographic, clinical, and morphometric factors. Associations are presented as Spearman’s correlations with shades of red indicating positive correlations and shades of blue indicating negative correlations.

In univariate analysis, having sarcopenia (yes/no) was associated with higher fasting glucose levels (*r* = 0.16, *p* = 0.04). Having myosteatosis (yes/no) or having lower skeletal muscle density were associated with higher fasting glucose (*r* = 0.26, *p* < 0.001), HbA1c levels (*r* = 0.33, *p* < 0.001), and c-peptide levels (*r* = −0.30, *p* = 0.02). In contrast, lower liver density associated with higher fasting insulin (*r* = −0.31, *p* < 0.001) and HOMA-IR score (−0.30, *p* < 0.001) as well as fasting glucose, HbA1c, and c-peptide levels (Fig. 1). Lower pancreas density also associated with higher fasting insulin (*r* = −0.16, *p* = 0.03) and HOMA-IR score (r = −0.15, *p* = 0.04) along with higher HbA1c (*r* = −0.19, *p* = 0.01).

**Fig. 2:**
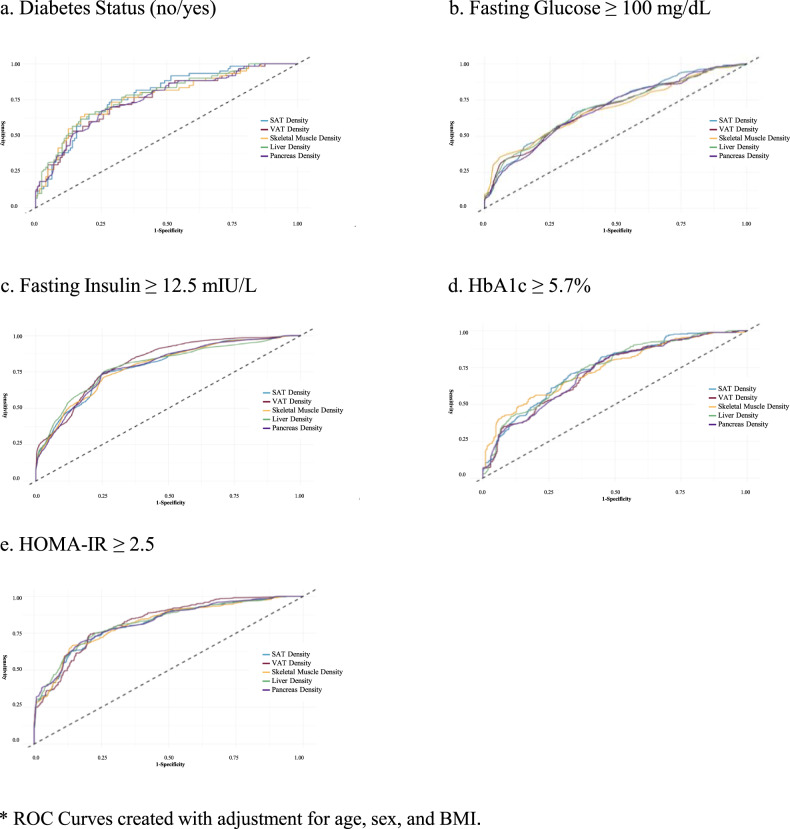
Comparisons of receiver operating curves for CT-quantified densities for abdominal subcutaneous adipose tissue, visceral adipose tissue, abdominal skeletal muscle, liver and pancreas. Each figure (**a**–**e**) presents a different glycemic/insulinemic outcome.

ROC curves (Fig. 2) adjusted for age, sex and BMI showed that the five tissue densities had similar ability to discriminate diabetes status as well as glycemic/insulinemic status when biomarkers were dichotomized. Comparison of AUC values (Table 2) confirmed the ability to identify participants at risk for each outcome was similar among depots with AUC scores ranging from 0.76 to 0.78 for diabetes status, 0.79 to 0.81 for fasting insulin, 0.82–0.83 for HOMA-IR score, and the differences in AUC between densities being 0.2 or less.

**Table 2:** Comparisons of areas under the receiver operating curves for CT-quantified densities of abdominal subcutaneous adipose tissue, visceral adipose tissue, abdominal skeletal muscle, liver and pancreas as predictors of five outcomes of glycemic/insulinemic status.

**Area under the ROC curve for diabetes status (no/yes)**	
SAT density	0.78
Liver density	0.78
SM density	0.77
VAT density	0.76
Pancreas density	0.76
**Area under the ROC curve for fasting glucose** **≥** **100** **mg/dL**	
SAT density	0.71
Liver density	0.69
SM density	0.69
VAT density	0.69
Pancreas density	0.69
**Area under the ROC curve for fasting insulin** **≥** **12.5 mIU/L**	
SAT density	0.79
Liver density	0.79
SM density	0.79
VAT density	0.81
Pancreas density	0.79
**Area under the ROC curve for HbA1c** **≥** **5.7%**	
SAT density	0.74
Liver density	0.74
SM density	0.74
VAT density	0.72
Pancreas density	0.72
**Area under the ROC curve for HOMA-IR** **≥** **2.5**	
SAT density	0.82
Liver density	0.82
SM density	0.82
VAT density	0.83
Pancreas density	0.82

Area under the ROC curve after adjustment for age, sex, and BMI.

Multivariable logistic regression modeling was adjusted for age, sex and BMI to assess the robustness of correlations between tissue densities and glycemic/insulinemic biomarkers. Upon adjustment, SAT density was associated with diabetes status and HbA1c, whereas VAT density was associated with fasting insulin level and HOMA-IR score (Supplemental Table). Further, in determining the model with the best overall fit for each biomarker (Table 3), higher SAT density was significantly associated with diabetes status and higher glucose and HbA1c levels. Lower liver density is significantly associated with diabetes status and higher HbA1c, whereas lower skeletal muscle density is associated with higher glucose and insulin levels.

**Table 3:** Best fitting logistic regression models of CT-quantified morphometric features for glycemic/insulinemic status.

Outcome = diabetes (no/yes)			Nagelkerke *R*^2^ = 0.40		
Predictors	Estimate	Std. error	Wald statistic	*p* value	95% CI
(Intercept)	10.191	3.760	7.346	0.007	
SAT area	0.007	0.002	15.28	<0.001	1.003–1.010
VAT area	0.005	0.002	4.61	0.03	1.000–1.009
SAT density	0.141	0.034	17.36	<0.001	1.078–1.231
Liver density	−0.056	0.021	7.19	0.007	0.907–0.985
Age	0.063	0.020	9.42	0.002	1.023–1.108

Outcome = glucose ≥ 100 mg/dL			Nagelkerke *R*^2^ = 0.29		
Predictors	Estimate	Std. error	Wald statistic	*p* value	95% CI
(Intercept)	7.627	3.076	6.15	0.01	
SAT area	0.003	0.002	3.50	0.06	1.000–1.006
VAT area	0.009	0.002	16.64	<0.001	1.005–1.014
SAT density	0.094	0.031	9.22	0.002	1.034–1.168
SM density	−0.062	0.029	4.36	0.03	0.888–0.994
Pancreas density	0.043	0.019	4.80	0.03	1.004–1.084

Outcome = insulin ≥ 12.5 mIU/L			Nagelkerke *R*^2^ = 0.44		
Predictors	Estimate	std. error	Wald statistic	*p* value	95% CI
(Intercept)	−11.152	2.624	18.06	<0.001	
SAT area	0.005	0.003	2.47	0.12	0.999–1.010
VAT area	0.012	0.003	13.38	<0.001	1.007–1.018
SM density	−0.072	0.035	4.22	0.04	0.885–0.997
BMI	0.185	0.075	6.07	0.01	1.039–1.395

Outcome = HbA1c ≥ 5.7			Nagelkerke *R*^2^ = 0.33		
Predictors	Estimate	Std. error	Wald statistic	*p* value	95% CI
(Intercept)	8.546	3.443	6.16	0.01	
SAT area	0.005	0.002	11.20	<0.001	1.002–1.009
VAT area	0.003	0.002	2.26	0.13	0.999–1.007
SAT density	0.114	0.032	13.07	<0.001	1.054–1.192
Liver density	−0.043	0.019	5.016	0.03	0.922–0.995
Age	0.062	0.018	12.27	<0.001	1.027–1.101

Outcome = HOMA-IR ≥ 2.5			Nagelkerke *R*^2^ = 0.42		
Predictors	Estimate	Std. error	Wald statistic	*p* value	95% CI
(Intercept)	4.255	3.814	1.25	0.27	
SAT area	0.011	0.003	14.00	<0.001	1.005–1.017
VAT area	0.015	0.003	22.79	<0.001	1.009–1.021
SAT density	0.082	0.042	3.70	0.05	0.998–1.179

## Discussion

In the present study, we determined differences in physiologic and cardiometabolic biomarkers between PWH based on glycemic status (normoglycemia, prediabetes, T2DM). To the best of our knowledge, this is the first evidence utilizing CT imaging to consider both quantity and quality of abdominal organs and skeletal muscle as factors associated with glucose intolerance and insulin resistance by comparing their morphometric features (amounts and densities) in PWH by glycemic status. While differences between those with versus without diabetes for glycemia, demographics, and more typical clinical biomarkers of cardiometabolic risk (i.e., age, BMI, waist circumference, triglycerides, TG/HDL ratio) mirrored what has been shown in the general population, we identified unique relationships between CT-measured morphometrics and biomarkers of glucose intolerance and insulin resistance. Interestingly, the densities of SAT, VAT, liver, pancreas, and skeletal muscle (reflecting the degree of lipid accumulation) were differentially associated with indicators of glycemic/insulinemic status—suggesting that the deposition of ectopic fat in various organs and tissues may be driven, at least in part, by disparate underlying physiological processes that lead to a common metabolic dysfunction endpoint.

It has been established that PWH are predisposed to excess weight gain, which may be an effect of the interaction between adipose tissue as a reservoir for HIV and the effects of some ART agents [25]. In the setting of obesity, HIV, and ART, pathophysiologic dysregulation within SAT to store excess energy as triglycerides results in the accumulation of ectopic fat in the liver, pancreas, other organs, and skeletal muscle [8]. Indeed, SAT in PWH shows enrichment of CD8^+^ and pro-inflammatory CD4^+^T cells, which contribute to fibrosis and impaired lipid storage [26]. As expected, we detected markedly increased SAT and VAT areas in participants with HIV and T2DM. Surprisingly, SAT density was highest in PWH and T2DM, while we observed no significant differences in VAT density or VAT:SAT ratio among the three groups. Our finding of higher SAT density in PWH and T2DM differs from much of the available research on newly diagnosed individuals with HIV [9, 22], and supports the hypothesis that changes secondary to the presence of HIV and effects of treatment may promote abnormally high SAT fibrosis. Consistent with this hypothesis, adipose tissue samples from PWH who have obesity show smaller adipocytes and 2.4-fold greater fibrosis in SAT compared to persons without HIV [27]. While higher adipose density should signify metabolically healthier tissue, SAT fibrosis associated with some ART agents, lipid engorgement of adipocytes, and the chronic inflammatory environment seen in HIV may explain the increased SAT density observed in our cohort of PWH and T2DM [26, 28, 29]. Interestingly, we observed a relationship between SAT density with glucose and HbA1c levels, as well as overall diabetes status, while VAT density was associated with insulin levels and HOMA-IR scores. The fact that ectopic fat in SAT and VAT contributes differently to cardiometabolic dysfunction may be related to variances in the production of adipokines. Analysis of SAT and VAT samples from bariatric surgery patients showed a similar divergence with interaction between SAT adipocyte size, HbA1c, and resistin, whereas VAT adipocyte size interacted with insulin and adiponectin [30]. Notably, dysregulated adipokine production contributes to both insulin resistance and pancreatic beta cell dysfunction [31].

Another novel distinction among participants categorized by glycemic status is that those with diabetes had significantly lower pancreas, liver, and skeletal muscle densities. Regarding skeletal muscle density, the incidence of myosteatosis in participants with diabetes was twofold higher than that of those with prediabetes and sevenfold higher than that of those with normoglycemia. Increased intermuscular adipose tissue reduces skeletal muscle insulin sensitivity, likely by secretion of adipokines and cytokines that promote a local inflammatory environment, disrupting lipolysis [32, 33]. Moreover, higher intramyocellular lipid has been associated with more severe insulin resistance [34]. Prior studies have shown that skeletal muscle density declines more rapidly in PWH, and having myosteatosis may be a precursor to developing sarcopenia, which has long-term adverse effects on physical strength and functional independence, and increases risk for mortality [16, 17]. Of concern, a recent study using artificial intelligence (AI) methods showed myosteatosis is a stronger predictor of mortality in asymptomatic adults than sarcopenia or hepatic steatosis [35].

Although evidence linking skeletal muscle myosteatosis with hepatic steatosis has been shown in some populations, it has been unclear whether lipid deposition in skeletal muscle and liver reflects distinctive metabolic derangements in PWH [36]. Although the prevalence of hepatic steatosis was 2.5-fold greater in participants with diabetes, we detected no significant relationship between skeletal muscle density and liver density in participants with or without diabetes. This finding is consistent with our prior work showing skeletal muscle and liver densities associate with different SAT adipocyte and lipid metabolism gene expression profiles [37]. Interestingly, we observed a weak relationship between SAT and skeletal muscle densities in contrast to a robust relationship between SAT and liver densities. However, AUC values showed liver and skeletal muscle densities were similar in their ability to discriminate participants based on dichotomized biomarkers of glycemic and insulinemic status, but regression modeling showed liver density was more significantly associated with having higher glucose levels or HbA1c, but did not significantly associate with insulin levels or HOMA-IR score.

Nonetheless, differences in the characteristics of skeletal muscle and liver densities support the construct that relationships with glycemic status in PWH are more complicated than only being a defect of adipose tissue with regard to energy homeostasis. For example, the severity of insulin resistance may differ between muscle and hepatic tissue, as hepatic insulin resistance may be more associated with extracellular remodeling and fibrosis [38]. Of note, the accumulation of ectopic fat in the liver progresses to end-organ damage as metabolic-associated fatty liver disease (MAFLD), which is more prevalent in PWH and almost triples the risk for T2DM [39]. While likely bidirectional, insulin resistance is a hallmark of both MAFLD and T2DM. Notably, insulin resistance in skeletal muscle and liver associates with different fasting and postprandial plasma metabolites [40, 41], as well as having different SAT RNA transcriptome profiles [42], suggesting at least partly distinct pathologies.

The reduced density of the pancreas that we observed in participants with T2DM may be a consequence of prolonged exposure to ectopic lipid, which would be consistent with being in a more advanced stage of cardiometabolic dysfunction [43]. A marked reduction in β-cell function, indicated by reduced insulin disposition index, has been reported in PWH who have altered adipose tissue distribution, with defective first-phase insulin secretion after glucose bolus [44, 45]. In other persons predisposed to T2DM, β-cell lipotoxicity has been evident with lipid infusion producing reduced first- and second-phase insulin secretion [46]. However, it should be noted that the findings showing high prevalence of low organ and tissue densities and relationships with biomarkers of glycemic/insulinemic status are not merely aging-related. While the age of participants with diabetes averaged approximately 10 years older than those without prediabetes or T2DM, and age was significantly associated with all outcomes, only 9% of all participants were over age 65 years. Intriguingly, VAT, skeletal muscle and pancreas densities were associated with age, whereas SAT and liver densities were not.

A key strength of the present study is that the three groups were well-matched for sex, BMI, and HIV specific factors, which enables determining differences specific to body composition/morphometrics that associate with biomarkers of glycemic/insulinemic status. Another major strength is the use of CT imaging to quantify both quantity (amount) and quality (density) of tissues, and particularly the segmentation of all five ectopic fat depots (SAT, VAT, liver, pancreas, and skeletal muscle) that are the most influential in the development of impaired glucose uptake and insulin action. However, one limitation is that very small differences in tissue density may not be easily detectable in standard imaging. Another limitation of the study is the cross-sectional nature, which prohibits causal inferences and determining temporal changes in body composition/morphometrics. Additionally, the low proportion of females, which reflects our patient population, was too small to make comparisons by sex.

CT imaging is a common clinical diagnostic tool that can also hold a novel and vital role in screening for and predicting degenerative health conditions. Using morphologic markers of quantity and quality of VAT, SAT, skeletal muscle, and organs such as the liver and pancreas enables detecting metabolic perturbations that may be occurring earlier than the onset of clinical symptoms that present once overt diabetes is present. Although we do not know the true directionality and it is also possible that these metabolic changes are a consequence of impaired glucose/insulin dynamics. Of serious concern is that for PWH, having diabetes reduces the survival rate by threefold compared to individuals without diabetes [46]. Given the high burden of comorbid conditions seen among the HIV+ population, it would be highly valuable to have a comprehensive identification of the variety of factors that increase risk and create more holistic personalized treatment plans to improve health and health outcomes.

## Supplementary information

Supplementary Table

## Data Availability

The statistical code and data are available upon reasonable request to the corresponding author.
