# Supplementary material for: Density of ectopic fat depots predict distinct biomarkers of glycemic and insulinemic status in persons with HIV
Source: Nutr Diabetes. 2025 Jun 18;15:28. doi: 10.1038/s41387-025-00381-y (PMC12177074; doi:10.1038/s41387-025-00381-y)
Supplement: Supplementary file 1 — Supplementary Table [file 41387_2025_381_MOESM1_ESM.docx]

Supplemental Table. Comparison of Multivariable Regression Models for CT-Quantified Densities as Predictors of Five Outcomes of Glycemic/Insulinemic Status in Persons with HIV

a. Outcome = Diabetes (no/yes) b. Outcome = Fasting Glucose ≥ 100 mg/dL

c. Outcome = Fasting Insulin ≥ 12.5 mIU/L d. Outcome = HOMA-IR ≥ 2.5

e. Outcome = HbA1c ≥ 5.7%
